# What makes *Komagataella phaffii* non-conventional?

**DOI:** 10.1093/femsyr/foab059

**Published:** 2021-11-25

**Authors:** Özge Ata, Burcu Gündüz Ergün, Patrick Fickers, Lina Heistinger, Diethard Mattanovich, Corinna Rebnegger, Brigitte Gasser

**Affiliations:** Department of Biotechnology, Institute of Microbiology and Microbial Biotechnology, University of Natural Resources and Life Sciences Vienna (BOKU), Muthgasse 18, 1190 Vienna, Austria; Austrian Centre of Industrial Biotechnology (ACIB), Muthgasse 11, 1190 Vienna, Austria; UNAM-National Nanotechnology Research Center, Bilkent University, Ankara, Turkey; Biotechnology Research Center, Ministry of Agriculture and Forestry, Ankara, Turkey; Microbial Processes and Interactions, TERRA Teaching and Research Centre, Gembloux Agro-Bio Tech, University of Liège, Av. de la Faculté 2B, 5030 Gembloux, Belgium; Department of Biotechnology, Institute of Microbiology and Microbial Biotechnology, University of Natural Resources and Life Sciences Vienna (BOKU), Muthgasse 18, 1190 Vienna, Austria; Austrian Centre of Industrial Biotechnology (ACIB), Muthgasse 11, 1190 Vienna, Austria; Christian Doppler Laboratory for Innovative Immunotherapeutics, University of Natural Resources and Life Sciences (BOKU), Muthgasse 18, 1190 Vienna, Austria; Department of Biotechnology, Institute of Microbiology and Microbial Biotechnology, University of Natural Resources and Life Sciences Vienna (BOKU), Muthgasse 18, 1190 Vienna, Austria; Austrian Centre of Industrial Biotechnology (ACIB), Muthgasse 11, 1190 Vienna, Austria; Department of Biotechnology, Institute of Microbiology and Microbial Biotechnology, University of Natural Resources and Life Sciences Vienna (BOKU), Muthgasse 18, 1190 Vienna, Austria; Austrian Centre of Industrial Biotechnology (ACIB), Muthgasse 11, 1190 Vienna, Austria; Christian Doppler Laboratory for Growth-Decoupled Protein Production in Yeast, University of Natural Resources and Life Sciences Vienna (BOKU), Muthgasse 18, 1190 Vienna, Austria; Department of Biotechnology, Institute of Microbiology and Microbial Biotechnology, University of Natural Resources and Life Sciences Vienna (BOKU), Muthgasse 18, 1190 Vienna, Austria; Austrian Centre of Industrial Biotechnology (ACIB), Muthgasse 11, 1190 Vienna, Austria; Biotechnology Research Center, Ministry of Agriculture and Forestry, Ankara, Turkey

**Keywords:** *Komagataella phaffii*, non-conventional yeast, biotechnology, protein production, carbon metabolism, methylotrophy

## Abstract

The important industrial protein production host *Komagataella phaffii* (syn *Pichia pastoris*) is classified as a non-conventional yeast. But what exactly makes *K. phaffii* non-conventional? In this review, we set out to address the main differences to the ‘conventional’ yeast *Saccharomyces cerevisiae*, but also pinpoint differences to other non-conventional yeasts used in biotechnology. Apart from its methylotrophic lifestyle, *K. phaffii* is a Crabtree-negative yeast species. But even within the methylotrophs, *K. phaffii* possesses distinct regulatory features such as glycerol-repression of the methanol-utilization pathway or the lack of nitrate assimilation. Rewiring of the transcriptional networks regulating carbon (and nitrogen) source utilization clearly contributes to our understanding of genetic events occurring during evolution of yeast species. The mechanisms of mating-type switching and the triggers of morphogenic phenotypes represent further examples for how *K. phaffii* is distinguished from the model yeast *S. cerevisiae*. With respect to heterologous protein production, *K. phaffii* features high secretory capacity but secretes only low amounts of endogenous proteins. Different to *S. cerevisiae*, the Golgi apparatus of *K. phaffii* is stacked like in mammals. While it is tempting to speculate that Golgi architecture is correlated to the high secretion levels or the different N-glycan structures observed in *K. phaffii*, there is recent evidence against this. We conclude that *K. phaffii* is a yeast with unique features that has a lot of potential to explore both fundamental research questions and industrial applications.

## Teaser

Non-conventional features render the yeast *K. phaffii* an attractive model organism and an efficient host for biotechnology applications.

## INTRODUCTION

During the course of evolution, many different lifestyles emerged among yeasts. Thanks to their diversity, the world is surrounded by distinct yeast species inhabiting many different environments, metabolizing several carbon sources and producing a variety of metabolites (Kurtzman, Fell and Boekhout 2011). *Komagataella phaffii* (formerly known as *Pichia pastoris*) is among the Ascomycota yeasts from the Saccharomycetes class (Heistinger, Gasser and Mattanovich 2020). Approximately, 5200 genes are encoded on four rather large chromosomes, with a total genome size of 9.4 Mb. *Komagataella phaffii* is a methylotrophic yeast that can utilize methanol as the sole carbon and energy source. Besides methanol, it can grow on a number of carbon sources including glucose, glycerol, ethanol, trehalose, L-rhamnose, mannitol, sorbitol, D-glucitol, lactic acid, succinic acid, acetic acid and citric acid (varies among different strains; Sreekrishna *et al*. 1997; Kurtzman, Fell and Boekhout 2011; Sahu and Rangarajan 2016).

Since the 1990s, *K. phaffii* is among the preferred hosts for recombinant protein production, and more recently, *K. phaffii* has also been employed for non-protein products (Peña *et al*. 2018; Werten *et al*. 2019; Zhu *et al*. 2019; Karbalaei, Rezaee and Farsiani 2020; Duman-Özdamar and Binay 2021; Gao, Jiang and Lian 2021). Furthermore, *K. phaffii* serves as a model organism in biomedical research and basic cell biology (Bernauer *et al*. 2020). Its attractivity as a biotechnological host and model organism is strongly connected to features that distinct *K. phaffii* from other yeasts and classify it as a non-conventional yeast.

## METHYLOTROPHY

The most obvious non-conventional feature of *K. phaffii* is its ability to metabolize methanol. Recent phylogenetic analyses of the budding yeasts show that all methylotrophic yeasts cluster in one clade (Shen *et al*. 2018), indicating that methylotrophy has probably evolved only once. The first enzymatic step of methanol utilization (MUT), the oxidation of methanol to formaldehyde, is catalyzed by an alcohol oxidase (Aox1/2). Sequence similarity proposes that this enzyme belongs to the group of glucose–methanol–choline (GMC) oxidoreductases (Ozimek, Veenhuis and van der Klei 2005). Different to other methylotrophic yeasts, *K. phaffii* possesses two genes encoding AOX, which are 97% identical on the amino acid level and form a 600-kDa homo-octamer containing eight FAD cofactors (Vonck, Parcej and Mills 2016; Ito *et al*. 2007). Formaldehyde is then assimilated by a transketolase, forming dihydroxyacetone and glyceraldehyde-3-phosphate from xylulose-5-phosphate and formaldehyde. This enzyme called dihydroxyacetone synthase is believed to have evolved from transketolase, however, having gained the ability to accept formaldehyde as an acceptor substrate (Kato *et al*. 1982). Xylulose-5-phosphate is recycled by sugar phosphate interconversions. In principle, all enzymes necessary to perform this recycling are present in the pentose phosphate pathway (PPP) of yeasts, as well as the reactions for the dissimilation of formaldehyde to CO_2_, by which *K. phaffii* generates energy. Evolution of methylotrophy, however, modified also the spatial distribution of the pathway enzymes to peroxisomes, creating methanol assimilation organelles. Rußmayer *et al*. (2015) showed that the entire xylulose monophosphate (XuMP) cycle for methanol assimilation is localized in the peroxisomes. Instead of using the canonical cytosolic PPP enzymes, the XuMP cycle is catalyzed by a set of isoenzymes (Das1/2, Fba1-2, Rki1-2, Rpe1-2 and Tal1-2), encoded by duplicated gene copies with peroxisomal targeting signals. This leads to the speculation whether the peroxisomal localization is an essential feature to enable methylotrophy in yeasts. This idea is supported by the fact that, despite substantial effort it has not been successful so far to create methylotrophy in bakers yeast *Saccharomyces cerevisiae* by simply transferring alcohol oxidase and dihydroxyacetone synthase to it. It may well be that the other peroxisomal pathway enzymes were missing, or specific features of peroxisomes that methylotrophic yeasts have likely evolved. With a transposon tagged knockout strategy, Zhu *et al*. (2018) have searched for the essential gene set that is specific for growth on methanol. The annotation of this gene set is poor, but could be improved by a meta-analysis with the functional annotation by Valli *et al*. (2016). Methanol-only essential genes included the expected pathway enzyme genes, a set of genes responsible for peroxisomal protein targeting, as well as genes encoding peroxisomal structural proteins. As all these genes were not essential for growth on glucose it can be speculated that they have a different function, are less abundantly expressed or are absent in non-methylotrophic yeasts. Espinosa *et al*. (2020) have recently reported that *S. cerevisiae* could be evolved to assimilate methanol in addition to glucose. While the exact pathway remains unresolved, these data indicate (1) that a basic enzymatic pattern for MUT is encoded in the budding yeast genome, and (2) that the native enzymes are not sufficient (at least in their localization) to support growth, so that the uniqueness of the methanol pathway in methylotrophic yeasts is underlined.

## CRABTREE PHENOTYPE AND DIFFERENT PATHWAYS FOR CARBON UTILIZATION

**Figure 1.**
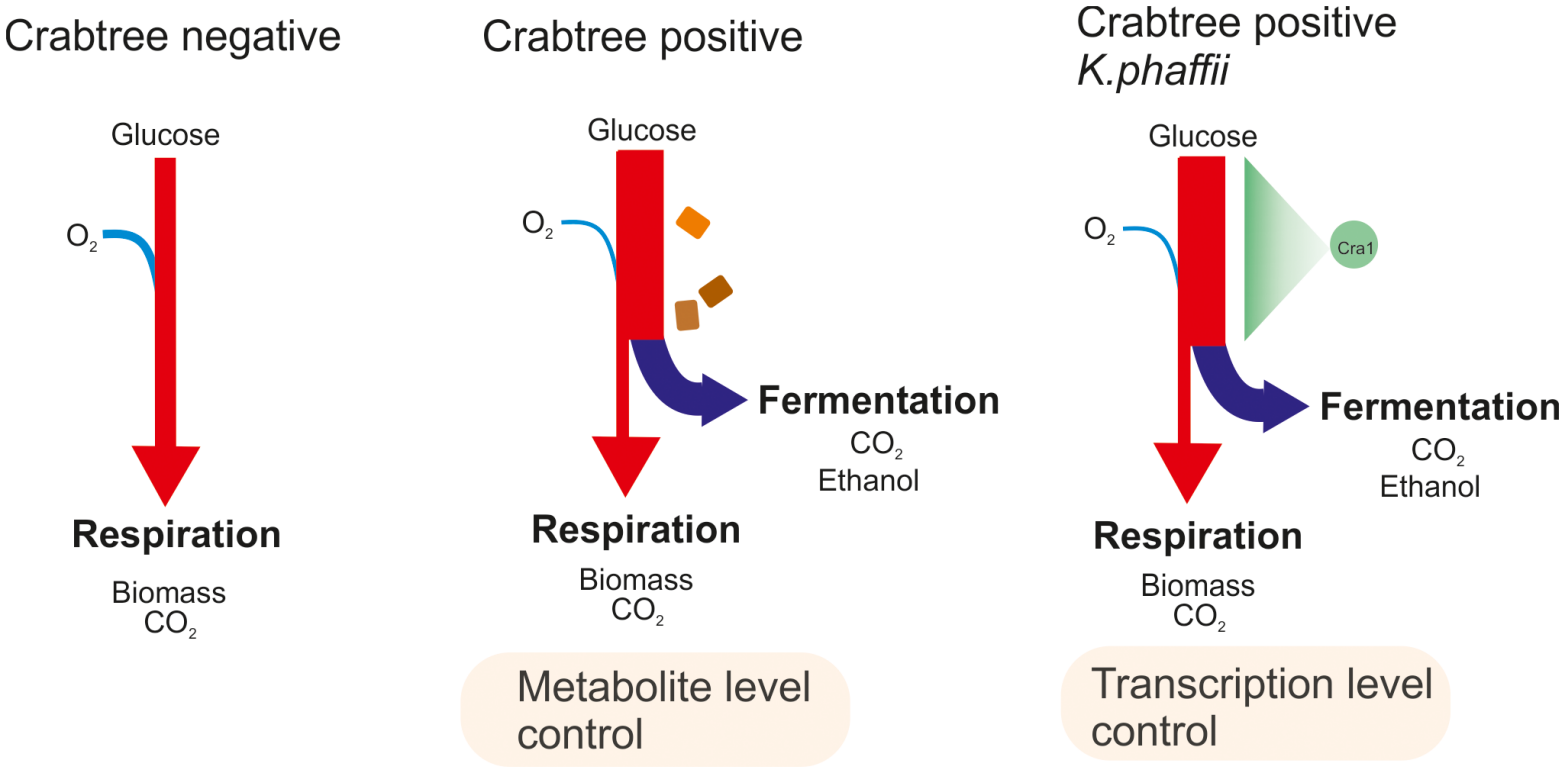
Regulation of the Crabtree phenotype in yeasts. (Left) Crabtree negative yeasts have a limited glucose uptake capacity and exclusively ferment under aerobic conditions. (Middle) Due to the overflow metabolism, Crabtree positive yeasts have a respiro-fermentative metabolism which is controlled at the metabolite level. (Right) Overexpression of *CRA1* upregulates the glycolytic genes leading to overflow metabolism, and demonstrating that glycolysis in *K. phaffii* is controlled at the transcriptional level.

Glucose is one of the carbon sources on which *K. phaffii* can readily grow and form biomass (Kurtzman, Fell and Boekhout 2011). However, glucose uptake is limited compared to Crabtree-positive yeasts such as *Saccharomyces cerevisiae* (q_Smax_ ≈ 0.35–0.60 g_S_/g_X_/h for *K. phaffii* and 2.88–2.16 g_S_/g_X_/h for *S. cerevisiae* in aerobic cultures (Diderich *et al*. 1999; Otterstedt *et al*. 2004; Maurer *et al*. 2006; Ata *et al*. 2018). It is argued that the high number of hexose transporters might be one of the reasons for the superior glucose uptake metabolism of Crabtree-positive (respiro-fermenting) yeasts. Accordingly, *S. cerevisiae* possesses more than 15 hexose transporters which allows it to transport glucose at a high rate (Boles and Hollenberg 1997; Elbing *et al*. 2004). Eventually, the high glucose flux exceeds the respiration capacity and leads to overflow metabolism of glycolysis which results in reduced biomass yields and ethanol production even at aerobic conditions (Fig. 1). In contrast, *K. phaffii* has a reduced number of hexose transporters similar to other respiratory yeasts such as *Kluyveromyces lactis*, *Hansenula polymorpha* (syn *​​Ogataea polymorpha and Ogataea parapolymorpha*) and *Scheffersomyces* (*Pichia*) *stipitis* (Mattanovich *et al*. 2009). It possesses one high affinity transporter (*GTH1*) and three *HXT* isozymes (*HXT1*, *HXT2* and *HXT400*; Valli *et al*. 2016). This prevents the overflow metabolism of glycolysis so that *K. phaffii* exclusively performs respiration under fully aerobic conditions (Ata *et al*. 2018). Therefore, it is classified as canonical Crabtree-negative yeast. Additionally, the fraction of carbon entering the PPP is higher in *K. phaffii*, in contrast to *S. cerevisiae* where the main flux is through glycolysis (see below). Recently, a transcription factor (TF), *CRA1* (a Gal4-like TF, homolog of Sc*GAL4*) was identified to be controlling glycolysis and fermentation metabolism of *K. phaffii* (Ata *et al*. 2018). Constitutive overexpression of this TF resulted in increased glucose uptake and ethanol production rates, as well as upregulation of glycolytic genes, which switched the Crabtree-negative phenotype of *K. phaffii* to Crabtree-positive (Fig. 1). Interestingly, the hexose transporters were not regulated by *CRA1* overexpression suggesting that hexose transporters are not responsible for the increased glycolytic flux in *K. phaffii*.

*Komagataella* *phaffii* cannot metabolize galactose but according to sequence homology it contains a *GAL10* gene in addition to *CRA1* (homolog of *ScGAL4*; Valli *et al*. 2016). In a comparative genomics analysis, it was shown that the ability of galactose utilization has been lost at least seven times during yeast evolution (Riley *et al*. 2016). It might be expected that a few *GAL* homologs in *K. phaffii* might have been inherited from an ancestral yeast that could consume galactose and eventually were lost during its evolution. The function of *GAL10* in *K. phaffii* is not known, but the Sc*GAL4* homolog *CRA1* is apparently controlling glycolysis in *K. phaffii*, in contrast to *S. cerevisiae* where Gal4 regulates the *GAL* genes (Ata *et al*. 2018). The whole genome duplication (WGD) marks a transition in the role of Gal4 where its function switched from a generalist TF controlling glycolysis to a specialist TF regulating galactose metabolism (Choudhury and Whiteway 2018). In yeasts which originate prior to this transition like *Candida albicans*, *Debaryomyces hansenii*, or *Schizosaccharomyces pombe*, the Leloir pathway for galactose utilization is controlled by Cph1. In post-WGD yeasts, Cph1 was eventually lost, Mig1 and Gal4 (along with other regulatory proteins) have been recruited to co-regulate the Leloir pathway, while in pre-WGD yeasts like *K. phaffii* and *C. albicans*, Gal4 has a regulatory function in the central carbon metabolism (Martchenko, Levitin and Whiteway 2007; Askew *et al*. 2009; Ata *et al*. 2018).

There is a high diversity among different yeast species on the ability of growing on glycerol as the sole carbon source (Kurtzman, Fell and Boekhout 2011). Unlike *S. cerevisiae*, *K. phaffii* can grow on glycerol in minimal media without requiring any additional supplements (μ_max_ of 0.26/h, q_Glycerol_max_ = 0.37 g_S_/g_X_/h; Jahic *et al*. 2002). Possession of four H^+^/glycerol symporters in addition to a Fps1-type glycerol facilitator demonstrates the superiority of the glycerol uptake metabolism compared to many other yeasts (Lages, Silva-Graça and Lucas 1999; Mattanovich *et al*. 2009). Of these four H^+^/glycerol transporters, GT1 (encoded by *STL1-1*, *PP7435_Chr1-0321*) was found to be one of the factors affecting the crosstalk between the glycerol and methanol metabolism in *K. phaffii* (Zhan *et al*. 2016). While the deletion of GT1 (*STL1-1*) did not cause a significant growth impairment in glycerol-based medium, it relieved glycerol repression on P*_AOX1_*. Additionally, overexpression of *GT1* repressed *MXR1* and *AOX1* expression, whereas *MXR1* overexpression repressed *GT1* (Zhan *et al*. 2016, 2017; Li *et al*. 2018).

In addition to regulating the MUT pathway, glycerol metabolism in *K. phaffii* seems to have a global effect on NADPH balance. Metabolic flux analysis with ^13^C-labeled glycerol showed that the major cytosolic NADPH source might be the glycerol catabolic pathways (Tomàs-Gamisans *et al*. 2019). In contrast to the previous assumption that the oxidative branch of the PPP is the main source for cytosolic NADPH, it was demonstrated that the flux through the PPP is almost negligible. Furthermore, it was hypothesized that, among the different glycerol catabolic pathways (Klein *et al*. 2017), the NADP-dependent glycerol oxidation pathway is the major cytosolic NADPH source in glycerol grown cultures. However, PPP seems to have a more profound effect on NADPH generation when the cells are grown on glucose: The split ratio of PPP flux is around 40–55% in *K. phaffii* (Baumann *et al*. 2010; Nocon *et al*. 2016; Ata *et al*. 2018) as opposed to *S. cerevisiae* which has a split ratio of 4–17% (Gombert *et al*. 2001; Maaheimo *et al*. 2001; Velagapudi *et al*. 2007).

L-rhamnose metabolism occurs in fungi *via* an oxidative (non-phosphorylated) pathway (Koivistoinen *et al*. 2012). A comparative genomics study showed that the genes of L-rhamnose metabolism are clustered among the yeasts analysed in the study (Riley *et al*. 2016). It appears that *K. phaffii* has five genes (*LRA1*, *LRA2*, *LRA3*, *LRA4* and *TRC1*) associated with L-rhamnose metabolism (Liu, Styles and Fink 2016; Valli *et al*. 2016). However, *PP7435_Chr1-0845 (LRA3)* does not cluster with the other genes, contrary to the gene arrangement in *S. stipitis. Komagataella phaffii* can grow on L-rhamnose as the sole carbon source with a slightly lower growth rate than on glucose and the expression of the related genes are induced by rhamnose and repressed in the presence of glucose (Liu, Styles and Fink 2016). 

## GENE REGULATION AND TFs

Methylotrophic yeasts offer a repertoire of regulated, strong promoters that are naturally regulating MUT pathway genes (Ergün *et al*. 2019b). Typically, the promoters of MUT pathway genes are tightly repressed on repressing carbon sources such as glucose and strongly induced when shifted to methanol. However, they demonstrate different modes of derepression among methylotrophic yeasts. The promoter of the *K. phaffii AOX1* gene P*_AOX1_* is strongly induced by methanol while repressed by ethanol, glucose and glycerol (Ergün *et al*. 2020). Derepressed *K. phaffii* cells (repressing carbon source is depleted or non-repressing carbon source is present) display approximately 2% *AOX1* transcriptional activity of the methanol-induced level, whereas methanol-induced cells display more than 1000-fold higher activity than fully repressed (glucose-grown) cells (Lin-Cereghino *et al*. 2006). In contrast, promoters of orthologous genes *AOD* (alcohol oxidase) in *Candida boidinii*, *MOX* (methanol oxidase) in *H. polymorpha* and *MOD1* (methanol oxidase 1) in *Pichia methanolica* show respectively 3–30%, 60–70% and 60–70% derepression in glycerol compared to their methanol-induced levels (Hartner and Glieder 2006). Substantial amounts of heterologous protein were expressed by P*_AOX1_* in *H. polymorpha* on glycerol, and P*_AOX1_* was regulated in the same manner as P*_MOX _*(Raschke *et al*. 1996). These findings suggest that rather than promoter *cis*-acting DNA elements, components of the cellular transcriptional machinery determine the expression mode of P*_AOX1_* on glycerol. Understanding the transcriptional regulation of P*_AOX1_* and other MUT pathway genes has paramount importance to enhance control over natural promoters and design new expression mechanisms in *K. phaffii*.

**Figure 2.**
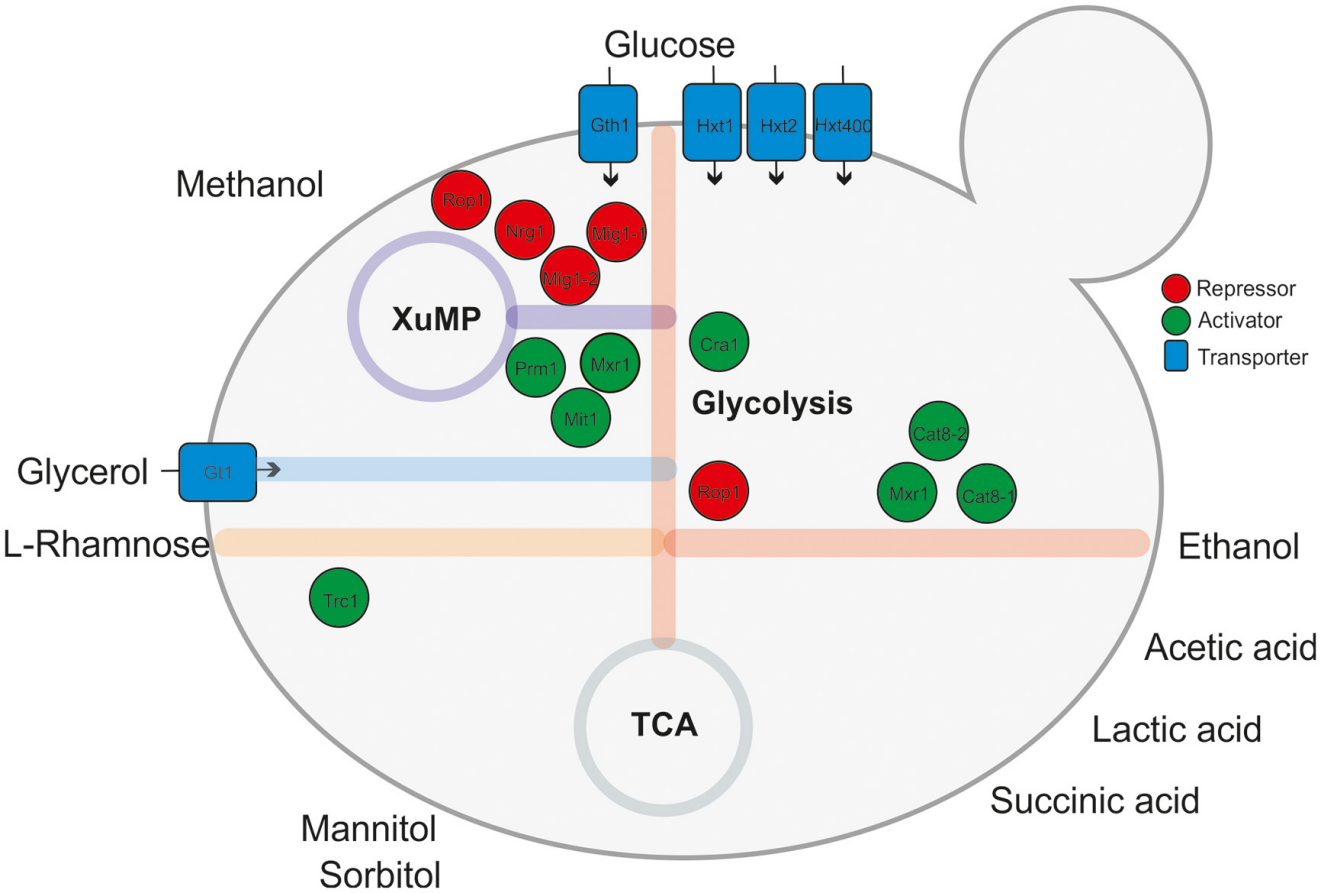
TFs involved in the regulation of *K. phaffii*’s central carbon metabolism. Blue rectangles represent the transporters. Activators and repressors are shown in green and red, respectively. See Table 1 for detailed information on the TFs. XuMP: Xylulose monophosphate pathway. TCA: Tricarboxylic acid cycle.

An overview of the characterized transcription factors (TFs) involved in regulation of carbon source utilization and their respective function in *K. phaffii* is given in Table 1 and Fig. 2.

**Table 1.** List of TFs that are experimentally confirmed to regulate carbon source utilization in *K. phaffii*.

TF	Uniprot ID*	Homologs in other yeasts	Function	References
Aft1	F2QPE8_KOMPC		Regulates genes of carbohydrate metabolism and recombinant protein secretion	Ruth *et al*. (2014)
Cat8-1	F2QS26_KOMPC	*S. cerevisiae* Cat8, *K. lactis* Cat8	Activates glyoxylate cycle and EUT pathway in ethanol grown *K. phaffii*, required for growth on acetate	Barbay *et al*. (2021)
Cat8-2	F2QYX3_KOMPC	*S. cerevisiae* Cat8/Sip4, *K. lactis* Cat8/Sip4	Activates carnitine shuttle and EUT pathway in ethanol grown *K. phaffii*	Barbay *et al*. (2021)
Cra1	F2QQF5_KOMPC	*S. cerevisiae* Gal4	Controls glycolysis and fermentation metabolism	Ata *et al*. (2018)
Flo8	F2QYE9_KOMPC	*S. cerevisiae* Flo8	Master regulator of filamentous growth and surface adherence, also involved in glucose repression	Rebnegger *et al*. (2016)
Mig1-1	F2QZJ1_KOMPC	*S. cerevisiae* Mig1, *H. polymorpha* Mig1	Repressor of MUT pathway and PEX genes	Wang *et al*. (2017); Shi *et al*. (2018)
Mig1-2	F2QPW6_KOMPC	*S. cerevisiae* Mig1, *H. polymorpha* Mig2	Repressor of MUT pathway and PEX genes	Wang *et al*. (2017); Shi *et al*. (2018)
Mit1	F2QV89_KOMPC	*H. polymorpha* Mpp1	Activator of MUT pathway but not PEX genes on methanol, represses P*_AOX1_* in response to glycerol	(Wang *et al*. (2016b)
Mxr1	F2QZ27_KOMPC	*S. cerevisiae* Adr1, *C. boidinii* Trm2	Activator of MUT pathway and PEX genes	Lin-Cereghino *et al*. (2006)
Trm1	F2QZY1_KOMPC	*C. boidinii* Trm1	Activator of MUT pathway and PEX genes	Sahu *et al*. (2014)
Trc1	F2QZI4_PICP7	*S. stipitis* Trc1	TF suggested to be involved in the regulation of *LRA* genes of the rhamnose metabolism	Liu, Styles and Fink (2016)
Nrg1	F2QUX2_KOMPC	*S. cerevisiae* Nrg1/2	Repressor of MUT pathway and PEX genes	Wang *et al*. (2016a)
Rop1	F2QW29_KOMPC		Repressor of MUT pathway, PEX genes and phosphoenolpyruvate carboxykinase	Kumar and Rangarajan (2012)

* For some TF genes different annotations are used in literature. In order to avoid ambiguity, their UniProt IDs are provided.

*S. cerevisiae* Adr1 (alcohol dehydrogenase synthesis regulator) has a pivotal role in the activation of glucose repressible genes, peroxisomal protein genes and ethanol, glycerol and fatty acid utilization pathway genes (Young *et al*. 2003).  Alcohol dehydrogenase Adh2 is the first enzyme of the ethanol utilization pathway that catalyzes oxidation of ethanol to acetaldehyde. Adr1 and Cat8 synergistically activate *S. cerevisiae ADH2* transcription and many other ethanol utilization pathway genes (Young *et al*. 2003). The *K. phaffii* Adr1-homologue Mxr1 (methanol expression regulator 1) has gained new functions and lost others through evolution as a result of changes in the environmental conditions, cell physiology and spectrum of genes that it controls. Deletion of *MXR1* caused total transcriptional shut down of *K. phaffii* P*_AOX1_* and cells cannot grow on methanol, while it had less detrimental effect on *K. phaffii* P*_ADH2_* activation and growth on ethanol compared to *S. cerevisiae* transcriptional regulation (Ergün 2018). *MXR1* is constitutively expressed at low levels and activates MUT pathway and peroxisome biogenesis (PEX) genes (Lin-Cereghino *et al*. 2006). Mxr1 is cytoplasmic in glucose-grown cells but localized to the nucleus in cells cultured on gluconeogenic substances (Lin-Cereghino *et al*. 2006). Similar to *S. cerevisiae* Adr1, the 14–3-3 protein directly interacts with Mxr1 by phosphorylation and inhibits its activity (Parua *et al*. 2012). The ethanol-repressible nature of P*_AOX1_* has been investigated through Mxr1, however, the answer came from a different side. Promoter engineering of P*_AOX1_* by introducing a Cat8 *cis*-acting DNA motif converted ethanol-repressible P*_AOX1_* to the ethanol inducible P*_AOX1/Cat8-L3_* variant (Ergün *et al*. 2020). This demonstrates that the ethanol repressible nature of P*_AOX1_* is due to the absence of an ethanol responsive *cis*-acting element. Addition of further Cat8 *cis*-acting motifs enhanced *K. phaffii* P*_ADH2_* expression 4.8-fold on ethanol (Ergün *et al*. 2019a), while the *K. phaffii Δcat8-1Δcat8-2* mutant lost its ability to grow on ethanol (Ergün 2018; Barbay *et al*. 2021), emphasizing the importance of the *K. phaffii* Cat8-1 and Cat8-2 TFs for ethanol regulation.

In the methylotrophic yeast *C. boidinii* Trm1 and Trm2 (transcriptional regulation of methanol induction) are essential TFs for the expression of MUT pathway and PEX genes (Sasano *et al*. 2008, 2010). Trm2 is the homologue of *K. phaffii* Mxr1 and responsible for the activation of methanol-inducible genes by relieving glucose repression, and also essential for Trm1-dependent gene activation (Sasano *et al*. 2010). *Komagataella phaffii* Trm1, the homologue of *C. boidinii* Trm1, is another positively acting TF of MUT pathway and PEX genes. The respective *Δtrm1* mutant showed impaired P*_AOX1_* activity and growth on methanol (Sahu, Krishna Rao and Rangarajan 2014), which could be rescued by *MIT1* overexpression (Wang *et al*. 2016b).

Mit1 (methanol-induced TF1) positively regulates MUT pathway genes, but not peroxisomal genes and activates P*_AOX1_* in response to methanol while it represses P*_AOX1_* transcription on glycerol (Wang *et al*. 2016b). *Komagataella phaffii Δmit1* cannot grow on methanol and P*_AOX1_* is not active. Complementation of the *Δmit1* mutant with *H. polymorpha* Mpp1 (methylotrophic peroxisomal protein) restored growth and P*_AOX1_* activity on methanol, and furthermore, lead to remarkable *AOX1* expression levels on glycerol (Wang *et al*. 2016b). Structural differences between Mit1 and Mpp1 likely contribute to the differential expression mode of P*_AOX1_* and P*_MOX_*.

Mxr1, Mit1 and Trm1 are binding to P*_AOX1_* at different sites and cooperatively activate P*_AOX1_*, but overexpression of Mit1 or Trm1 does not restore P*_AOX1_* activity in the *Δmxr1* mutant. Firstly, derepression of P*_AOX1_* is mediated by the master TF Mxr1, then Trm1 and Mit1 contribute to promoter activation (Wang *et al*. 2016b). *TRM1* is expressed constitutively in glucose, glycerol and methanol, while *MIT1* expression is strongly induced on methanol. Both Mit1 and Trm1 are localized to the nucleus in glucose, glycerol and methanol conditions. Mit1 binds to P*_AOX1_* in the presence of all three carbon sources, however, Trm1 only binds when cells are grown on methanol or glycerol but not on glucose (Wang *et al*. 2016b).

Rop1 (repressor of phosphoenolpyruvate carboxykinase; Kumar and Rangarajan 2012) and Nrg1 (Wang *et al*. 2016a) are negative regulators of *K. phaffii* MUT pathway and PEX genes. Rop1 and Mxr1 function antagonistically but exhibit the same DNA binding specificity whereby Rop1 binds to DNA with higher affinity than Mxr1 (Kumar and Rangarajan 2012). *S. cerevisiae* Nrg1 and Nrg2 (negative regulator of glucose-repressed genes) mediate glucose repression (Zhou and Winston 2001). *K*.*phaffii* Nrg1 represses MUT pathway and PEX genes in glucose and glycerol conditions. It directly binds to five positions on P*_AOX1_*, two of which overlap with MXREs (Wang *et al*. 2016a). The *Δnrg1* mutant showed a growth defect when cultivated on glucose, glycerol or methanol. Unexpectedly, overexpression of *NRG1* was found to enhance secretion of recombinant proteins through a yet unidentified mechanism (Stadlmayr *et al*. 2010).

*S. cerevisiae* Mig1 and Mig2 are involved in glucose repression of different carbon metabolism genes (Schüller 2003). *Komagataellaphaffii* Mig1-1 and Mig1-2 are mainly localized in the nucleus in glucose or glycerol conditions, while they translocate to the cytosol when cells are grown on methanol (Wang *et al*. 2017). Glycerol-induced suppression of P*_AOX1_* is partially removed in the *Δmig1-1* mutant, while no effect is observed in the *Δmig1-2* mutant. The double knock-out *Δmig1-1Δmig1-2* mutant showed increased transcriptional activation (Wang *et al*. 2017), which seems to be mediated through activation of Mit1 (Shi *et al*. 2018). On the other hand, neither deletion of *MIG1-1* nor *MIG1-2* deregulated P*_AOX1_* on glucose. The double deletion of *MIG1-1* and *MIG1-2* led to a growth defect on glycerol and glucose (Shi *et al*. 2018).

## MATING AND MATING-TYPE SWITCHING

*K. phaffii* is a preferentially haploid yeast usually propagating by mitotic cell division. Sexual reproduction (mating and spore formation) is possible but can only be observed under nitrogen starvation conditions (Feng *et al*. 2020). In contrast to *S. cerevisiae*, where haploid cells mate spontaneously to form stable diploids, the mating-type (*MAT*) genes and most other mating-relevant genes of *K. phaffii* are not expressed in rich medium (Heistinger, Gasser and Mattanovich 2018). Once mating has occurred, diploid *K. phaffii* cells rapidly undergo meiosis and sporulation if no selective pressure is applied. This coupling of mating and sporulation has also been observed in other yeasts like *Candida lusitaniae* or *K. lactis* but is absent in *Saccharomyces* species, where the lack of nitrogen and presence of a non-fermentable carbon source act as a trigger for sporulation of diploid cells (Booth, Tuch and Johnson 2010; Merlini, Dudin and Martin 2013; Sherwood *et al*. 2014; Hanson and Wolfe 2017).

**Figure 3.**
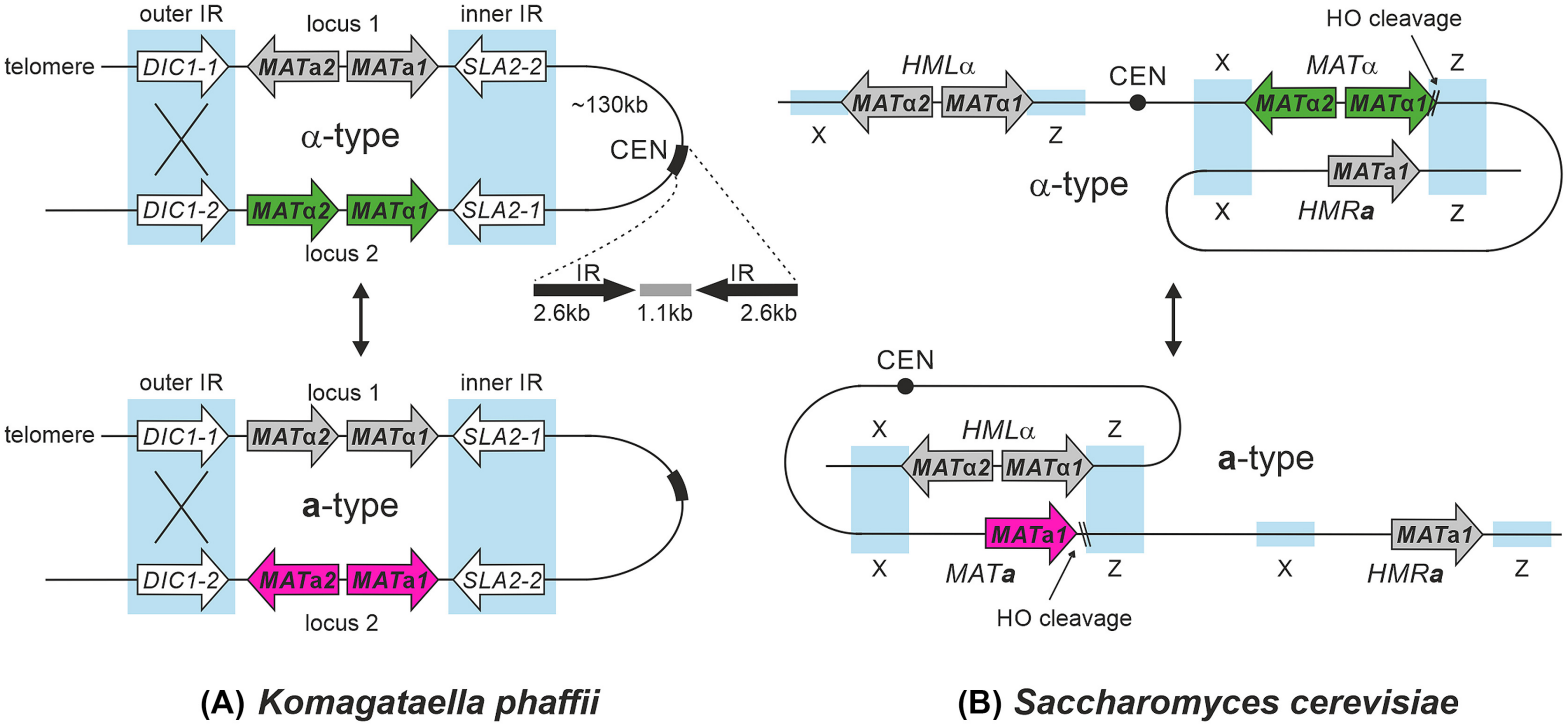
Mating-type systems of *K. phaffii* and *S. cerevisiae*. **(A)** Homologous recombination at the outer inverted repeat (IR) region results in mating-type switching by inversion of the genomic region between the two *MAT* loci, including the centromere of chromosome 4 in *K. phaffii*. **(B)** In *S. cerevisiae*, HO endonuclease initiates mating-type switching. The two silent *MAT* loci (*HML*α and *HMR***a**) serve as template for mating-typ switching *via* a synthesis dependent strand annealing mechanism. The concept to display the mating type switches is based on Hanson and Wolfe (2017).

Although the mating-type and mating behavior of yeasts is generally regulated by the *MAT* genes, there are significant differences in the mechanism of mating-type switching and the control of cell type regulations between different species. *K. phaffii* has a two-locus mating-type system, where both loci, containing either *MAT***a***1* and *MAT***a***2* or *MAT*α*1* and *MAT*α*2*, are located at the beginning of chromosome 4 (Fig. 3). The two loci are flanked by inverted repeat (IR) sequences containing one (*DIC1*) and three (*SLA2*, *SUI1* and *CWC25*) genes and are separated by around 135 kb of DNA sequence also containing the centromere (Hanson, Byrne and Wolfe 2014). Under mating conditions, the *MAT* locus next to the telomeric region remains silenced, while the genes in the second *MAT* locus are transcribed and thereby determine the mating-type of the cell (Heistinger, Gasser and Mattanovich 2018).

*K. phaffii* is a secondary homothallic yeast and mating-type switching takes place by homologous recombination at the ‘outer’ inverted repeat region containing the *DIC1* genes. This leads to an inversion of the whole chromosomal region between the two *MAT* loci and a swap of the *MAT* allele in the active mating-type locus (Hanson, Byrne and Wolfe 2014). A similar, well studied mating-type system is found in the methylotrophic yeast *O. polymorpha*, where the mating loci are flanked by one inverted repeat region and silencing of the silent *MAT* locus is mediated by its proximity to centromeric heterochromatin (Hanson, Byrne and Wolfe 2014; Maekawa and Kaneko 2014). A recent study analysing the *MAT* loci of more than 300 budding yeast species found that such two-locus flip-flop switching mechanisms have evolved independently at least ten times, while the three-locus mating type system as it is found in *S. cerevisiae* and closely related species seems to have evolved only once within the budding yeasts (Krassowski *et al*. 2019). In *S. cerevisiae*, two silent *MAT* loci (*HML*α and *HMR***a**) serve as template for mating-type switching via a synthesis dependent strand annealing mechanism. Switching is initiated by the HO endonuclease, which introduces a double strand break at the active *MAT* locus (Strathern *et al*. 1982; Ira, Satory and Haber 2006; Haber 2012; Hanson and Wolfe 2017). The genomes of methylotrophic yeasts do not contain orthologs of HO endonuclease and although it has been shown that nitrogen starvation induces switching via an Rme1 and Ste12 dependent pathway in *O. polymorpha*, it remains unclear by which molecular mechanism mating-type switching is initiated (Hanson, Byrne and Wolfe 2017; Yamamoto *et al*. 2017).

As described above, the Mat TFs encoded in the active *MAT* locus determine the mating-type of a cell. In *K. phaffii*, Mat**a**2 and Matα1 in **a**- and α-type cells, respectively, activate the expression of mating-type specific genes like the pheromone and pheromone surface receptor genes and are thereby essential for mating. In diploid cells, *MAT***a***1* and *MAT*α*2* are required for sporulation and the repression of mating (Heistinger, Gasser and Mattanovich 2018). In *S. cerevisiae* and other post-WGD yeasts, a rewiring of this regulatory network resulted in the loss of **a**-specific gene activation by Mat**a**2 and the requirement for **a**-specific gene repression by Matα2 in α-type cells (Tsong *et al*. 2006; Sorrells *et al*. 2015). 

Due to the requirements of prolonged nitrogen starvation and solid media, mating of *K. phaffii* is unlikely to occur under industrial production conditions, even though there are always cells of both mating-types present in the culture when working with homothallic strains. However, mating represents a useful tool for strain development as it can be applied to combine interesting traits, investigate genetic determinants of relevant traits by methods like quantitative trait loci (QTL) mapping or the generation of combinatorial libraries (Chen *et al*. 2012; Liti and Louis 2012; Swinnen, Thevelein and Nevoigt 2012). The use of heterothallic strains, such as the *K. phaffii* CBS2612 Δ*dic1-2* strains generated by the deletion of the ‘outer’ homologous region required for mating-type switching, further enables the use of *K. phaffii* for classical genetic studies (Heistinger, Gasser and Mattanovich 2018).

## CENTROMERES

The centromeres of *K. phaffii* consist of a 2–2.7 kb inverted repeat region with a central core sequence of around 1 kb. Each of the four chromosomes has one centromere which is unique in sequence. The centromere of chromosomes 3 and 4 are found close to one chromosome end, with the centromere on chromosome 4 being located within the invertible region flanked by the *MAT* loci (Figure 3). The centromeres were identified as large non-transcribed regions and replicate early during cell division (Coughlan *et al*. 2016; Sturmberger *et al*. 2016). A ChIP-seq experiment showed that the centromere-specific histone variant Cse4 is most abundant in the core sequence but its signal can be detected all along the non-transcribed centromeric region. The orientation of the centromeres is variable in different *K. phaffii* isolates, indicating that recombination at the inverted repeats can occur (Coughlan *et al*. 2016). The inverted repeat centromeres of *K. phaffii* are highly different from the small point centromeres found in *S. cerevisiae* and closely related species, which are only around 125 bp long and defined by a clear consensus sequence (Hegemann and Fleig 1993). Although inverted repeat centromeres are also found in other yeasts like *Candida tropicalis*, the small genome and efficient tool for manipulation make *K. phaffii* an interesting system to study centromere function (Chatterjee *et al*. 2016). So far, two studies have reported the characterization of *K. phaffii* plasmid vectors carrying whole centromeric sequences. Those plasmids were found to increase mitotic stability while maintaining a low copy number when compared to classical ARS plasmids (Nakamura *et al*. 2018; Piva *et al*. 2020).

## MORPHOLOGY SWITCHES

Upon experiencing adverse environmental conditions, budding yeasts can switch from unicellular to multicellular lifestyle, leading to flocculation, pseudohyphae formation or invasive growth (Brückner and Mösch 2012). These morphogenetic events give rise to subpopulations of cells exhibiting different phenotypes, thus providing advantage for adaptation to environmental changes and increasing chances of survival. In *S. cerevisiae* these morphology switches are associated with the flocculin (*FLO*) gene family, which has five dominant members encoding GPI-anchored cell-wall proteins (Verstrepen and Klis 2006; Willaert 2018). Out of these Flo1, Flo5, Flo9 and Flo10 are involved in flocculation, while Flo11 is responsible for filamentous growth (Guo *et al*. 2000; Van Mulders *et al*. 2009; Goossens and Willaert 2012). Most *S. cerevisiae* laboratory strains are devoid of flocculation and filamentous growth, as there is a defect in the *FLO8* gene encoding the master transcriptional activator (Liu, Styles and Fink 1996).

Morphological differentiations have also been observed in *Komagataella* species, however, until recently not much was known of the genetic and biochemical basis underlying these phenotypes. *K. phaffii* possesses an expanded *FLO* gene family consisting of 12 members containing different Flo-domains, and a Flo8-type TF (De *et al*. 2020). Pseudohyphae formation and surface adherence are absent when Flo8 is deleted, indicating that Flo8 is also the major regulator of filamentous growth in *K. phaffii* (Rebnegger *et al*. 2016; De *et al*. 2020). In both, *S. cerevisiae* and *C. albicans*, Flo8 was demonstrated to form a heterodimer with Mss11 *via* their N-terminal LisH domains that cooperatively regulates filamentous growth (Su *et al*. 2009; Kim *et al*. 2014). However, while also in *K. phaffii* Flo8 contains an N-terminal LisH domain no Mss11 homolog was identified (De *et al*. 2020), suggesting that Flo8 acts as a homodimer in this organism. While there is a clear ortholog of *FLO11*, for the other structural *FLO* genes no distinct homologs can be identified (Kock *et al*. 2018; Brückner *et al*. 2020; De *et al*. 2020). Kock *et al*. (2018) studied the nine predicted adhesins with a lectin-like PA14 domain present in *Komagataella* spp, and identified that the *Komagataella* adhesins form a unique clade in the fungal kingdom, thus stressing that it is hard to infer *FLO* gene function from one yeast species to another. Among them KpFlo1 (also named Cea1 or Flo5-2) was discovered as the first fungal adhesin showing high specificity for terminal β-GlcNAc capped glycans including chitinous polymers. The authors speculate that the *Komagataella* PA14 domain containing proteins evolved to adapt the cells to their specific habitat to govern cell-substrate interactions different from flocculation.

Only very little is known about the mechanisms leading to floc formation in *K. phaffii*. Flocculation is caused by homotypic cell-cell adhesion, whereby yeast cells aggregate into multicellular masses (flocs) that sediment out of the medium (Soares 2011). In *S. cerevisiae*, flocculation is triggered by carbon source limitation, pH variations, external stressors or the presence of ethanol or ions e.g. Ca^2+^ (Soares 2011). Cell–cell adhesion is depending on the N-terminal PA14 lectin domains present in *S. cerevisiae* Flo1, Flo5, Flo9 and Flo10 and their reversible binding of cell wall mannans (Goossens *et al*. 2015). The respective genes are located adjacent to telomeres, and are silenced through their subtelomeric localization during normal growth conditions (Soares 2011; Cullen and Sprague 2012). It is not known so far which members of the *FLO* gene family are responsible for flocculation in *K. phaffii*. Unlike in *S. cerevisiae*, no silencing of the subtelomeric *FLO* genes was observed in exponential growth conditions at pH 5.0 (De *et al*. 2020).

Mbawala *et al*. (1990) showed that cells exhibiting higher flocculation show elongated mannose chains containing alpha-1,2 and beta-1,2 linkages, indicating that also in *K. phaffii* lectin-like mechanisms are involved in this cell–cell adhesion process. Addition of 2 mM EDTA which captures Ca^2+^ involved in glycan cross-linking was reported to reduce floc formation observed during growth of *K. phaffii* in unbuffered YPD media (Tanneberger *et al*. 2007). In our experience, floc formation can be observed macroscopically at a pH around 4.0, and is reversible upon shifting the pH (De 2019).

Importantly, flocculation and sedimentation can be advantageous in industrial bioprocesses, as a rapid and efficient means of separation of the biomass from the product containing supernatant (e.g. in industrial ethanol fermentation processes of *S. cerevisiae*, (Soares 2011)). In this respect, *K. phaffiis* cells engineered for increased rhamnose metabolic flux were shown to exhibit strong flocculation and sedimentation in rhamnose-containing media (Yan *et al*. 2018).

**Figure 4.**
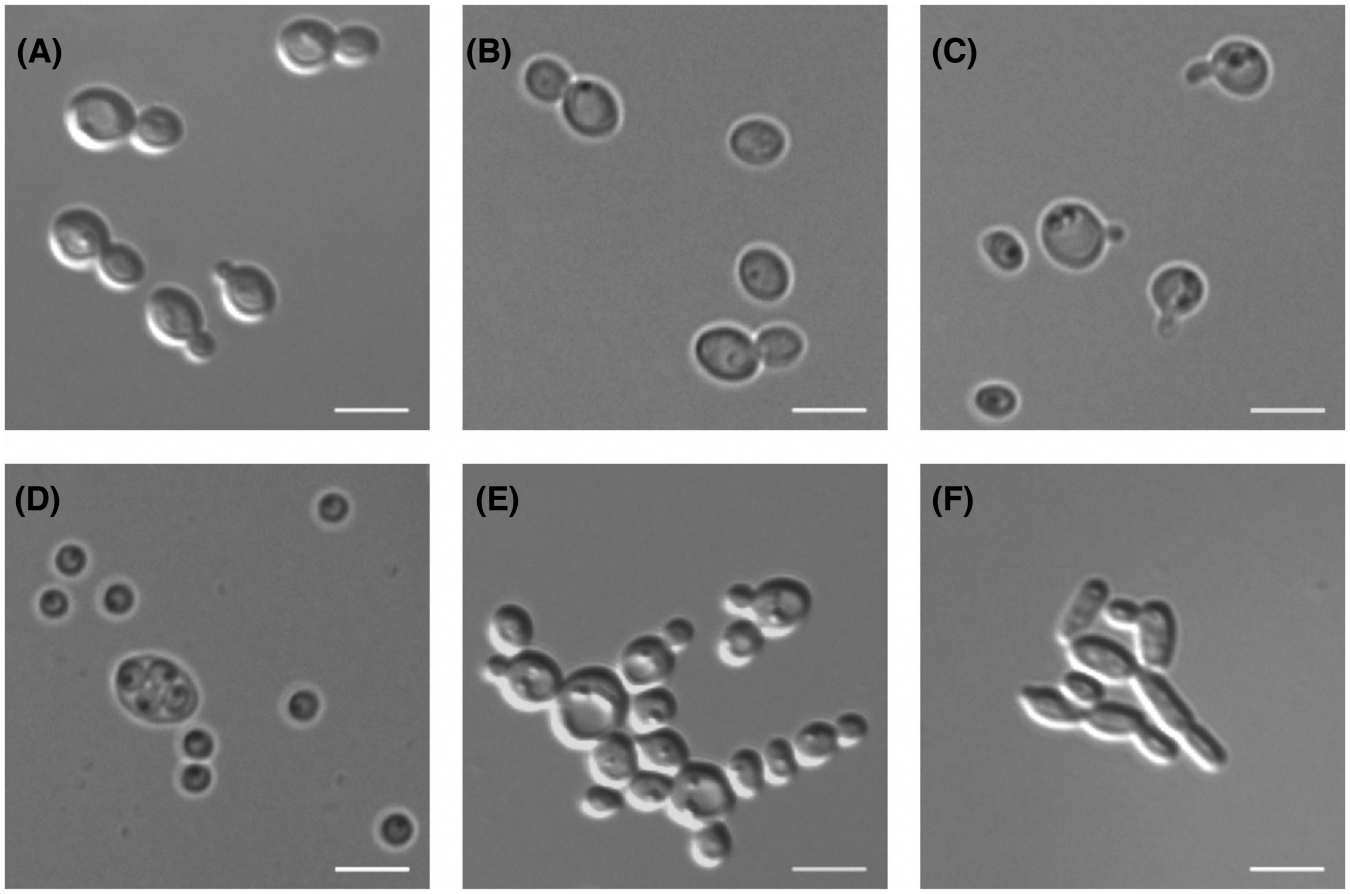
Diverse morphology of *K. phaffii*. **(A)** and **(B)** budding cells in rich medium, **(C)** shmoo formation, **(D)** sporulation, **(E)** flocculation and **(F)** pseudohyphal growth. Images were taken in brightfield or DIC mode. Scale bar 5 µm.

During pseudohyphal growth, filament-like structures are formed as cells divide but remain attached to each other (Cullen and Sprague 2012). If the filaments extend into a solid substrate, the phenomenon is termed invasive growth. In *S. cerevisiae*, pseudohyphal growth is dependent on Flo11 and more prevalent in diploid cells while invasive growth is more prevalent in haploid cells (Soares 2011; Cullen and Sprague 2012; Song and Kumar 2012). Despite being haploid, invasive growth has so far not been observed in *K. phaffii*. Also the environmental triggers leading to pseudohyphal growth are different between the two species: In contrast to *S. cerevisiae*, *K. phaffii* morphology is not affected by fusel alcohols or nitrogen starvation (De *et al*. 2020). So far, an elongated phenotype representing pseudohyphae was only observed when *K. phaffii* was cultivated at slow growth rates below *µ* = 0.075/h in glucose-limited chemostats (Rebnegger *et al*. 2014; De *et al*. 2020). The transition of *K. phaffii* from yeast to pseudohyphal form is associated with transcriptional changes of at least three *FLO* genes ( *FLO11*, *FLO400* and *FLO5-1*, all under control of Flo8) as well as chromatin remodeling. In contrast to *S. cerevisiae*, deletion of *FLO11* reduced but did not abolish pseudohyphae formation in *K. phaffii*. On the other hand, deletion of either *FLO400* or *FLO5-1* prevented the morphological changes. This was associated with a lack of *FLO11* induction upon switching to slow growth rates in glucose-limited chemostats, suggesting that *K. phaffii* Flo400 and/or Flo5-1 act as upstream signals for the activation of *FLO11*. However, it is not known which signaling cascades are responsible (De *et al*. 2020). Surprisingly, some strategies preventing morphological differentiations also resulted in higher productivity of secreted recombinant proteins (Gasser, Mattanovich and Buchetics 2014). Representative microscopic images of different morphological states of *K. phaffii* as described in this and preceding chapters are shown in Fig. 4.

## NITROGEN METABOLISM

Although nitrogen is an essential macronutrient for yeasts, its metabolism has been poorly investigated. Moreover, as most of available data concern *S. cerevisiae*, highlighting the non-conventional traits of *K. phaffii* is less obvious as compared to carbon metabolism for instance. In both *K. phaffii* and *S. cerevisiae*, ammonia and organic nitrogen are the main N-sources, and N-metabolism is mainly based on deamination and transamination reactions with ammonia, glutamate and glutamine as key compounds. From BLASTp searches (E value cut-off 10^–6^), all transporters for ammonia (three genes; Marini *et al*. 1997) and amino acids (21 genes; Bianchi *et al*. 2019) characterized so far in *S. cerevisiae* seem to have a counterpart in *K. phaffii* (data not shown). However, despite this similarity, it turned out that one needs to be careful by drawing conclusions from *S. cerevisiae*. For example, branched-chain amino acid (BCAA) biosynthesis is differentially compartmentalized in *K. phaffii*, with solely cytosolic α-isopropylmalate synthase Leu4 and BCAA aminotransferase Bat1 (Förster *et al*. 2014). Furthermore, enzymes involved in alanine (Alt1), aspartate (Aat2), glutamate (Gdh2, Gdh3 and Glt1) and lysine (Lys20 and Lys21) synthesis are found primarily in the cytosol of *K. phaffii*, whereas they localize also to the mitochondria in *S. cerevisiae* (Valli *et al*. 2020). In *S. cerevisiae*, two NADPH-glutamate dehydrogenases encoded by *GDH1* and *GDH3* catalyze the transamination of α-ketoglutarate with the formation of glutamate. *K. phaffii* possesses only one Gdh enzyme, termed Gdh3 (CP014584, with about 70% of identity in amino acid sequence to *S. cerevisiae* Gdh1 and Gdh3), similarly to *Yarrowia lipolytica* (Trotter *et al*. 2020) and *S. stipitis* (Freese *et al*. 2011). Glutamate can be further transaminated by glutamine synthase (gene *GLN1*) to yield glutamine. Inversely, ammonia can be released by deamination of glutamine or glutamate by glutamate synthase (gene *GLT1*) and NAD-glutamate dehydrogenase (gene *GDH2*), respectively (Magasanik 2003). These three key metabolites are the major precursors of amino acid biosynthesis while a-ketoglutarate is the hinge between C- and N-metabolisms.

In some methylotrophic yeasts such as *H. polymorpha*, nitrate assimilation occurs by its reduction into ammonium through the consecutive action of nitrate and nitrite reductases encoded by the genes *YNR1* and *YNL1*, respectively (Siverio 2002). This is not the case for *K. phaffii* that does not assimilate nitrate or possess nitrate reductase activity (Unkles *et al*. 2004). Even though the majority of media contain ammonium as nitrogen source, *K. phaffii* can also efficiently grow on urea at least in complex medium (Guo *et al*. 2012), and the addition of urea to the fermentation medium was discussed to be beneficial for protein production purposes (Adivitiya, Mohanty and Khasa 2021). As most of the other hemiascomycetes, *K. phaffii* contains a clear homolog of the bifunctional biotin-dependent urea amidolyase Dur1,2, but not nickel-dependent urease Ure1 or the corresponding Ni^2+^/Co^2+^ transporter Nic1 (Navarathna *et al*. 2010). Correspondingly, depletion of Co^2+^ from the medium had no effect on growth or productivity of *K. phaffii* (Pekarsky *et al*. 2020). Furthermore, there are up to five putative urea transporters found in the *K. phaffii* genome (four homologs of the *S. cerevisiae* Dur3 and one of *S. stipitis* Dur8), but nothing is known on their functionality or regulation so far (Valli *et al*. 2016, 2020). 

Some specificities of *K. phaffii* could be also highlighted from amino acid catabolic pathways. For instance, the asparagine degradation in *S. cerevisiae* is composed by a single copy *ASP1* gene and four repeated copies of *ASP2* encoding cytosolic and cell-wall asparaginases (League, Slot and Rokas 2012). These enzymes catalyze the deamination of asparagine into aspartate with the release of ammonium. From BLASTp analysis, no clear Asp2 counterpart could be identified in the *K. phaffii* genome, while sequence NC_012965.1 from strain GS115 shows 57% identity with Asp1 (data not shown).

By contrast to *S. cerevisiae, K. phaffii* and other non-conventional yeasts are able to grow in minimal medium containing aspartate or glutamate as sole N- and C-sources (Sahu and Rangarajan 2016). This ability has been found to be correlated to the activity of Gdh2. A *Δgdh2* mutant of *S. stipitis* cannot utilize glutamate as C-source while in *Y. lipolytica* the activity of Gdh2 increases up to 18-fold as compared to Gdh1 when glutamate is both, C- and N-source (Trotter *et al*. 2020). In *K. phaffii*, TFs that regulate methanol metabolism have also been found involved in this process. For instance, Mrx1 but not Trm1 or Rop1, regulates the activity of *GDH2* expression at post-transcriptional level. Mxr1 also regulates at the transcriptional level the genes *AAT1* and *AAT2* encoding mitochondrial and cytosolic aspartate aminotransferase, respectively, and the gene *GLN1*. Mxr1 Response Elements (MXREs) have been found in the promoter sequence of *AAT2* and *GLN1*. Therefore, methanol metabolism, a peculiar feature of *K. phaffii*, also controls N-metabolism. Beside this, nitrogen sources such as casamino acid have also been reported to regulate methanol metabolism (Velastegui *et al*. 2019). Indeed, expression of the genes *AOX1*, *DAS1* and *FLD1* is reduced in the presence of 0.1% of casamino acid.

Recently, a genomic survey of nitrogen assimilation pathways in budding yeast has been published (Linder 2019). By contrast to *S. cerevisiae*, the genome of *K. phaffii* contains the gene *AMO1* encoding amine oxidase that catalyzes the deamination of aliphatic primary amine (R-NH_2_) with the release of ammonia. It also contains the gene *AOC1* encoding lysyl oxidase. The corresponding enzyme has been biochemically characterized in detail (Kucha and Dooley 2001). Regarding uracil catabolism, *K. phaffii* genome contains genes *URC1* and *URC4* encoding putative cyclohydrolase and ribosyl-urea degrading enzymes which are missing in *S. cerevisiae*. In budding yeast, purines, uric acid and allantoin are all catabolized in a common pathway with ammonia as the final product. A total of 10 genes are involved in this pathway namely, *XAN1*, *XAN2*, *URO1*, *URO2*, *URO3*, *DAL1*, *DAL2*, *DAL3* and *DUR1,2*, although some of them are not present in the *S. cerevisiae* genome. These are the two *XAN* genes and the three *URO* genes that are putatively involved in the conversion of xanthine into uric acid and of uric acid into allantoin. Xanthine oxidoreductase *(XAN* genes) are known to require a molybdenum cofactor (MoCo) to be active (Mendel 2013). At least six genes, *MOC1*–*MOC6* are involved in molybdenum cofactor biosynthesis in eukaryotic cells. The homologs of these six genes were identified in *K. phaffii* but not in *S. cerevisiae* (Linder 2019).

## PROTEIN SECRETION

Based on the status of *K. phaffii* as a popular recombinant protein production platform, a large body of dedicated research aimed at elucidating (or manipulating) the molecular mechanics governing protein synthesis, secretion and post-translational modifications (PTMs) such as disulfide bond formation, proteolytic processing as well as N- and O-glycosylation. According to published literature, secretion yields of recombinant products produced in *K. phaffii* often exceed those of *S. cerevisiae* (e.g. Morton and Potter 2000; Dragosits *et al*. 2011; Tran *et al*. 2017). Similar to *S. cerevisiae*, about 10% of the total genes in the *P. pastoris* genome are predicted to have a function in the secretory pathway, including genes annotated to ER, protein folding, glycosylation, proteolytic processing, ERAD, Golgi, the vacuole, SNAREs and other genes involved in vesicle‐mediated transport (Delic *et al*. 2013). An extensive review comparing the canonical protein secretion pathway of seven different yeast species with that of *S. cerevisiae* on a genomic level was published by Delic *et al*. (2013), highlighting some noteworthy differences between the different species. One aspect that is specifically relevant to the production of biopharmaceuticals are differences in N- and O-glycosylation, as both types of PTM have been demonstrated to affect pharmacokinetics and pharmacodynamics of recombinantly produced proteins (De Wachter, Van Landuyt and Callewaert 2018; Zhou and Qiu 2019). N-glycosylation plays a very important role in the folding and quality control (QC) process of glycosylated proteins. The calnexin cycle, a crucial component of this QC process, is characterized in mammals and *S. pombe*. Homologs of the key enzyme UGGT (UDP-glucose:glycoprotein glucosyltransferase) also exist in several *Saccharomycetales* including *K. phaffii* and *Y. lipolytica*, but not in *S. cerevisiae* (Caramelo and Parodi 2008). Recently, it was demonstrated for *S. cerevisiae* that also O-glycosylation plays a decisive role in ER-quality control via a process termed unfolded protein O-mannosylation (UPOM), where proteins are only subjected to O-glycosylation if their correct confirmation is not attained for prolonged time periods. This increases their solubility and eventually leads to their degradation by the proteasome-dependent ERAD-pathway or, after exit from the ER, target them for post-ER degradation (Neubert *et al*. 2016).

Protein N-glycosylation in eukaryotes is initiated in the ER by the linking of the precursor Glc_3_Man_9_GlcNAc_2_ to an asparagine residue in the consensus sequence asparagine-X-serine/threonine (Asn-X-Ser/Thr, where X is any amino acid except for proline). Subsequently, the terminal α-1,2 and α-1,3 glucose residues are removed by respective glucosidases and one α-1,2-mannose is removed by an ER-residing α-1,2-mannosidase, resulting in an Man_8_GlcNAc_2_ glycan. Further N-glycan modifications of properly folded proteins take place in the Golgi apparatus, where yeasts add mannose and mannosylphosphate sugars to the Man_8_GlcNAc_2_ glycan core, generating N-glycans of the high-mannose type (Hamilton and Gerngross 2007; De Wachter, Van Landuyt and Callewaert 2018). N-glycan structure and side chain composition can differ substantially between yeast species (Thak *et al*. 2018) but can also be very heterogeneous in regard to a specific N-glycosylation site. *S*.*cerevisiae* N-glycans typically carry longer mannose outer chains, containing up to 150–200 mannose residues in total, while in *K. phaffii* outer mannose chain length is shorter with a total of 8–18 mannose residues (Herscovics and Orlean 1993; Kang *et al*. 1998; Krainer *et al*. 2013; Thak *et al*. 2018). Different to *S. cerevisiae*, *K. phaffii* glycans do not have immunogenic terminal α-1,3-linked mannose residues, due to the lack of the corresponding Mnn1 enzyme family (Delic *et al*. 2013; Thak *et al*. 2018). Instead, *K. phaffii* possesses Bmt enzymes which catalyse the addition of beta-1,2 mannoses.

Mannose outer chain elongation is initiated by the introduction of an α-1,6-mannose residue by the mannosyltransferase Och1. Disruption of this gene in *K. phaffii* leads to a reduction from 10 to 8 mannose residues in the dominant glycan (Krainer *et al*. 2013) and co-overexpression of a recombinant α-1,2-mannosidase in the ER mainly yields Man_5_GlcNAc_2_ structures (De Wachter, Van Landuyt and Callewaert 2018). In contrast, in *S. cerevisiae* two more enzymes acting on Man_8_GlcNAc_2_ were required to be deleted, which are not present in the *K. phaffii* genome. While it was initially believed that *K. phaffii* lacking Och1 shows only a minor phenotype in contrast to *S. cerevisiae* Δ*och1*, it was later elucidated that the true *K. phaffii* Δ*och1* knockout has a wrinkly morphology and a growth deficit (Krainer *et al*. 2013; De Wachter, Van Landuyt and Callewaert 2018), which was not observed in the initial insertional mutant (Choi *et al*. 2003; Vervecken *et al*. 2004). Nevertheless, in combination with the fact that less knockouts are required, this is probably one explanation why glycoengineering proceeded mainly with *K. phaffii* rather than *S. cerevisiae*. De Wachter, Van Landuyt and Callewaert (2018) comprehensively reviewed recent advances in N- and O-glycoengineering of yeasts. So far, most progress regarding the humanization of N-glycans has been made in *K. phaffii*, allowing even for the production of proteins containing complex-type sialylated N-glycans or mucin-type O-glycans. Nevertheless, to date no biopharmaceuticals produced in glycoengineered yeasts have reached the market.

In yeasts, O-glycosylation is also initiated in the ER by a family of protein O-mannosyltransferases (PMTs) that transfer mannose from dolichol phosphate β-D-mannose (Dol-P-Man) to Ser/Thr residues in the nascent proteins. Linear elongation of the O-glycans takes place in the Golgi, where mannosyltransferases catalyze the transfer of mannose from GDP mannose (Neubert *et al*. 2016). As true for many other genes, the number of PMT genes is reduced to five in *K. phaffii* compared to seven in *S. cerevisiae*. Similar to other yeasts, *K. phaffii PMT1* and *PMT2* appear both to have a predominant role in protein O-glycosylation, with *PMT2* showing the highest expression levels and the *Δpmt2* mutant strain the most severe growth defect (Govindappa *et al*. 2013; Nett *et al*. 2013). Radoman *et al*. (2021) comprehensively analysed the occurrence and composition of O-glycans of secreted proteins that were produced in *K. phaffii*, and found that the degree of O-mannosylation of a recombinant protein proved to be higher when methanol was used as a carbon source. The majority of O-glycans was composed of one mannose residue, while a maximum of five mannose residues was observed. It is not known, however, which enzymes are responsible for chain elongation, as *K. phaffii* possesses six homologs of the Golgi-resident Ktr/Kre-family mannosyltransferases compared to nine family members in *S. cerevisiae*, making their correct functional assignment as well as their disruption more difficult in *K. phaffii*. 

Major differences between *S. cerevisiae* and non-conventional yeasts are seen in the structural organization of the Golgi apparatus, which consists of disk-shaped membranes called cisterna. Golgi resident proteins, mainly involved in N- and O-glycan as well as lipid processing, are spatially organized, depending on their respective function. Freshly synthesized proteins that pass the ER-quality control, exit the ER *via* COPII-coated transport vesicles at so called transitional ER (tER) sites and enter the Golgi at the *cis* site, move through medial cisternae and eventually arrive at the *trans* compartment (Papanikou and Glick 2009; Suda and Nakano 2011). In most eukaryotes, Golgi cisternae form stacks. In vertebrates, the structural organization of the Golgi is even more complex, appearing as a twisted, ribbon-like network (Wei and Seemann 2010). *S. cerevisiae* is one of only a few known eukaryotic organisms where the respective Golgi compartments are not organized in stacks but scattered across the cytoplasm (Mowbrey and Dacks 2009). *K. phaffii* on the other hand, contains 2–5 stacks of ca. 4 cisternae each per cell and shares several other Golgi characteristics with mammalian cells such as the presence of a cisternae-surrounding matrix as well as fenestration and tubular extension of the cisternae (Papanikou and Glick 2009). It, therefore, serves as a model organism for Golgi-related research. Disruption of Golgi stacking in mammalian cells has been demonstrated to reduce total N-linked protein glycosylation and decrease N-glycan complexity. Reversible unstacking of the Golgi in *K. phaffii* was observed in a *SEC16* temperature sensitive mutant (Connerly *et al*. 2005) as well as the KO mutants of *PpLMH1*, encoding a GRIP domain Golgin (Jain, Dahara and Bhattacharyya 2019). Permanent unstacking under physiological conditions was achieved by the disruption of the genes *RSN1*, *CSC1-2* or *TVP18*, which are thought to be involved in calcium transport or signaling. However, unstacking of the Golgi by disruption of these genes did not lead to significant changes in the cellular N-glycome nor in N-glycan abundance or composition of glycoGFP (Aw *et al*. 2021).

One of the major advantages attributed to *K. phaffii* is that it secretes comparatively few host cell proteins, facilitating down-stream processing, and thereby reducing production costs. According to Lum and Min (Lum and Min 2011) the predicted secretome size of *S*.*cerevisiae* is 50%, and that of *Y. lipolytica*, another popular host for recombinant protein production, is even 300% larger than that of *K. phaffii*. However, these numbers do not account for growth conditions or for proteins regularly observed extracellularly such as metabolic enzymes, chaperones or proteins involved in translation that are not actively secreted but reach the extracellular space either by passive transport, unconventional secretion or cell lysis (Nombela, Gil and Chaffin 2006; Miura and Ueda 2018). Several studies have been conducted in order to characterize the secretome of *K. phaffii* under industrially relevant conditions (Dragosits *et al*. 2009; Huang *et al*. 2011; Burgard *et al*. 2020). The most recent study carried out by Burgard *et al*. (2020) investigated how the secretome of *K. phaffii* changes throughout a typical recombinant protein production process and how the choice of carbon source (glucose or glycerol/methanol) affects native protein secretion. In total 51 proteins were identified, concordant with previous observations. A similar study conducted in *K. lactis*, whose predicted secretome size varies between 113 and 178 proteins (Swaim *et al*. 2008; Lum and Min 2011), found up to 120 proteins when cells were grown on galactose and a total of 151 proteins across all growth conditions. In both, *K. lactis* and *K. phaffii*, a majority of identified proteins possessed a predicted signal peptide. While the core secretome (proteins identified in every condition) of *K. phaffii* mainly consisted of cell wall proteins, in *K. lactis* also many proteins with a function in glycosylation, carbohydrate metabolism and proteolysis were identified. To the authors knowledge no studies investigating the full secretome of other important yeast recombinant protein production hosts like *S. cerevisiae*, *H. polymorpha* or *Y. lipolytica* under industrially relevant conditions have been published so far, hindering relevant comparisons to these organisms.

## CONCLUSIONS

Genetic diversity is large among budding yeasts. Their morphological similarity should not make us believe that they function very similarly. Partly, differences between *S. cerevisiae* and non-conventional yeasts can be explained by the WGD event and subsequent functional specialization that *S. cerevisiae* and its close relatives went through. Non-conventional species such as *K. phaffii* have rather adapted their proteome to the ecological niches they habitate. But even where gene sets are similar, different transcriptional control creates multitute. Such regulatory differentiations have recently been identified in carbon metabolism and contribute to the different physiology of *K. phaffii* in comparison to *S. cerevisiae*. Also, differential localization of proteins was observed. It remains to be identified in future if such regulatory differences also contribute to the higher secretion efficiency and different glycan pattern that make *K. phaffii* an excellent protein production host. Overall, we strongly recommend being careful when drawing analogies solely based on sequence analysis.

## FUNDING INFORMATION

BG, OA and DM acknowledge funding by the Austrian BMK, BMDW, SFG, Standortagentur Tirol, Government of Lower Austria, Vienna Business Agency and BOKU through the FFG—COMET Funding Program. OA acknowledges funding by the Austrian Science Fund FWF (grant number M2891). BG, LH, DM and CR acknowledge funding by the Austrian BMDW and Nationalstiftung FTE through the Christian Doppler Research Association. This article is based upon work from the Yeast4Bio COST Action 18229, supported by COST (European Cooperation in Science and Technology).
